# TINNET COST Action BM1306: an international standard for outcome measurements in clinical trials of tinnitus

**DOI:** 10.1186/1745-6215-16-S1-P18

**Published:** 2015-05-29

**Authors:** Deborah Hall, Alain Londero, Winifred Schlee

**Affiliations:** 1 National Institute for Health Research (NIHR) Nottingham Hearing Biomedical Research Unit, Nottingham, NG1 5DU, UK; 2 University of Nottingham, NG7 2RD, UK; 3 Hôpital Européen Georges-Pompidou, Paris 5 University, France

## Background

Over 70 million people in Europe experience tinnitus, for 7 million it creates a debilitating condition. In spite of its enormous socioeconomic relevance, research funding is somewhat limited. The European Union has approved funding for a COST Action TINNET (2014-2018) to create a pan-European tinnitus research network. One of the Working Groups will address outcome measurement; building upon the 2006 consensus meeting organised by the Tinnitus Research Initiative [1]. This Working Group seeks to embrace inclusivity and brings together clinicians, experts on clinical research methodology, statisticians, and representatives of the health industry. The primary objective is to establish an international standard for outcome measurements in clinical trials of tinnitus.

## Methods

The first step towards the objective is to seek a consensus about appropriate and relevant outcome domains, using Delphi survey methodology. Details of the study design and collaborative approach will be confirmed at the first Working Group management team on November 14^th^. On November 13^th^, we are also holding a COST Action workshop in Amsterdam, “Agreed Standards for Measurement : An International Perspective” with invited talks on the COSMIN and the HOME initiatives, and the World Health Organisation International Classification of Functioning, Disability and Health (ICF) core sets for assessment of hearing loss.

## Conclusion

Once our methodology is confirmed we will register our work on the COMET database. Furthermore, by working with the COST Action Clinical and Database Working Groups we can achieve standards for outcome measurement both in clinical trials and in clinical routine and support data collection of treatment results in a centralised database.
